# Interleukin 22 attenuated angiotensin II induced acute lung injury through inhibiting the apoptosis of pulmonary microvascular endothelial cells

**DOI:** 10.1038/s41598-017-02056-w

**Published:** 2017-05-19

**Authors:** Zhiyong Wu, Zhipeng Hu, Xin Cai, Wei Ren, Feifeng Dai, Huagang Liu, Jinxing Chang, Bowen Li

**Affiliations:** Department of Cardiovascular Surgery, Renmin Hospital of Wuhan University, Jiefang Road 238, Wuhan, 430060 China

## Abstract

Apoptosis of pulmonary microvascular endothelial cells (PMVECs) was considered to be closely related to the pathogenesis of acute lung injury (ALI). We aim to investigate whether IL-22 plays protective roles in lung injury through inhibiting the apoptosis of PMVECs. ALI model was induced through subcutaneous infusion of angiotensin II (Ang II). Lung injury and infiltration of inflammatory cells were evaluated by determining the PaO_2_/FiO_2_, calculation of dry to weight ratio in lung, and immunohistochemisty analysis. Apoptosis of PMVECs was determined using TUNEL assay and flow cytometry, respectively. Immunofluorescence and Western blot analysis were used to determine the expression and localization of STAT3, as well as the nucleus transmission of STAT3 from cytoplasm after IL22 treatment. Pathological findings showed ALI was induced 1 week after AngII infusion. IL22 inhibited the AngII-induced ALI, attenuated the edema in lung and the infiltration of inflammatory cells. Also, it contributed to the apoptosis of PMVECs induced by AngII. Meanwhile, significant increase was noticed in the expression of STAT3, phosphorylation of Y705-STAT3, and migration from cytoplasm to the nucleus after IL-22 treatment (P < 0.05). The activation of STAT3 by IL22 showed significant attenuation after AG490 treatment. Our data indicated that IL22 showed protective effects on lung injury through inhibiting the AngII-induced PMVECs apoptosis and PMVEC barrier injury by activating the JAK2/STAT3 signaling pathway.

## Introduction

Acute aortic dissection (AAD) is frequently reported to occur accompanied by acute lung injury (ALI) featured by severe oxygenation impairment in lung^1, 2^. Our previous study reported elevation of Angiotensin II (Ang II) in AAD patients, especially those combined with ALI. In these patients, Ang II induced pulmonary microvascular endothelial cells (PMVECs) apoptosis, which subsequently resulted in interruption of the integrity of the PMVEC barrier^3^.

IL-22, a member of the IL-10 cytokine family^4, 5^, has been reported to be closely related to the pathogenesis of certain diseases^6^. Previously, IL-22 was reported to involve in several biological processes such as protecting tissues from damage, contributing to tissue repair and maintenance of tissue integrity, modulating innate immunity, and promoting anti-microbial defenses^7–10^. Unlike most of the other interleukins, IL-22 does not participate in regulation of immune cells function^7^. Instead, it targets cells at outer-body barriers, including the skin and tissues of the digestive and respiratory systems, as well as cells of the pancreas, liver, kidney and joints. Recently, several studies have been performed to address the roles of IL-22 in ALI^11, 12^. This leads us to investigate the roles of IL-22 in the pathogenesis of AAD complicated with ALI.

Although little is known about the biological roles of IL-22 in PMVECs and endothelial barrier function, several studies have demonstrated the beneficial effects of IL-22 on endothelial cells. In 2006, Chang *et al*. showed the expression of IL-22R was elevated in endothelial cells^13^. Besides, Shang *et al*. revealed IL-22 contributed to the viability, activation and angiogenesis of human umbilical vein endothelial cells (HUVECs)^14^. On this basis, we speculated that IL-22 may involve in the anti-apoptosis of PMVECs, which may be related to the protective effects in lung.

In this study, mice ALI model was established through subcutaneous injection of AngII, and then the roles of IL-22 in the lung injury and the PMVEC barrier were investigated. Besides, IL-22 was used to treat the cultured PMVECs to investigate its roles in the anti-apoptosis of PMVECs.

## Materials and Methods

### Establishing of AngII-induced ALI model in mice

ALI model was induced through subcutaneous infusion of Ang II. Male mice (8 weeks old) purchased from HFK Bioscience Co., Ltd (C57BL/6 J, Beijing, China) were fed on a normal diet, combined with osmotic mini pumps (Alzet, Cupertino, CA) filled with AngII (1 μg/kg per minute, Sigma-Aldrich) for 1 week (AngII group) or 0.9% normal saline (1 μl/kg per minute) for 1 week (sham group). Animals fed on a normal diet serves as control. All animal handling was performed in accordance with the Wuhan Directive for Animal Research and the current Guidelines for the Care and Use of Laboratory Animals published by the National Institutes of Health. Mice were sacrificed after anesthesia using 10% chloral hydrate (250 mg/kg) via intraperitoneal injection. Extensive measures were taken to minimize the sufferings of the animals. The study protocols were approved by the Ethical Committee of the Renmin Hospital of Wuhan University.

### Confirmation of IL22 roles in animal models

To investigate the protective roles of IL-22 in lung, peritoneal injection of IL-22 (20 μg/Kg, CYT-173, ProSpec) was administrated every two days. Besides, AG490 (10 mg/Kg, sc-202046, Santa Cruz) was given to inhibit the activity of JAK2 every two days via peritoneal injection.

### Cell culture

Rat PMVECs purchased from Bena Culture Collection (category No. BNCC338210, Suzhou, China) were cultured using the standard method as previously described^15^. Briefly, cells were cultured in endothelial culture medium (No. 1001, Sciencell) containing 5% fetal bovine serum (FBS, No. 0025), 1% endothelial cell growth supplement (No. 1052) and 1% penicillin/streptomycin solution (No. 0503) in 5% CO_2_ at 37 °C. PMVECs (P2-4) were expanded in monolayers in cell culture bottle. The culture medium was changed to a serum-free solution for 24 h prior to usage. The cells were divided into four groups, including (i) control group; (ii) AngII group, treated by 1 μM AngII (Sigma-Aldrich, St. Louis, USA); (iii) AngII+IL-22 group, treated by 1 μM AngII and 20 ng/mL IL-22 (CYT-173, ProSpec, CA, USA); and (iv) AngII+IL-22+AG490 group, treated by AngII (1 μM)+ IL-22 (20 ng/mL) and AG490 (10 µM, sc-202046, Santa Cruz, CA, USA). The cells were incubated for 48 h before cellular apoptosis assay.

### Blood gas analysis

To assess the pulmonary function, blood gas analysis was performed in subsets of experiments by obtaining arterial blood. Upon intraperitoneal injection of 10% chloral hydrate (250 mg/kg), the left ventricle was exposed and the arterial blood was obtained via cardiac puncture. The analysis was performed immediately after blood sample collection with an GEM Premier 3500 blood gas analyzer (Instrumentation Laboratory Co., Hartwell Avenue Lexington, MA,USA). The arterial partial pressure of oxygen was measured to calculate the PaO_2_/FiO_2_.

### Histopathological examination

The lung tissues were fixed and embedded, followed by cutting into sections (4 µm). Afterwards, HE staining and immunohistostaining were performed to determine the expression of CD68 (No. 333801, Biolegend, San Diego, CA, USA) and MPO (66177-1-Ig, Proteintech, Wuhan, China) according to the previous descriptions^16, 17^. Finally, the images were observed using a BX51 light microscope (Olympus Corporation, Tokyo, Japan).

### Expression and localization of STAT3 and pY705-STAT3

After antigen recovery, the primary antibodies (STAT3, category No. ab68153, Abcam, CA, USA; pY705-STAT3, category No. ab202876, Abcam, CA, USA) were added and co-incubated at 4 °C overnight. After labeling and incubating with Cy3 (No. BA1032, Boster Co., Ltd. Wuhan, China) at 37 °C, the expression of STAT3 was determined using an Eclipse 80i fluorescence microscope (Nikon, Tokyo, Japan).

### Western blotting

Total protein was extracted from the lung tissues in mice, and the total protein and nucleoprotein were extracted from the PMVECs. The resultant protein concentrations were determined by BCA Protein Assay reagents (Beyotime Biotechnology, Jiangsu, China) according to the previous description^18^. The expression of STAT3, pY705-STAT3, and Bcl-2 was determined using a standard protocol. The transferred membrane was blocked with 10% skimmed milk for 1 h at room temperature, and then incubated with the primary antibodies against STAT3 (1:1000; Abcam), and pY705-STAT3 (1:700; Abcam), Bcl-2 (1:700; Santa Cruz), and β-actin (1:500; Santa Cruz) overnight at 4 °C, respectively. After incubating with the horseradish peroxidase-conjugated secondary antibodies (1:5000; Zhong Shan-Golden Bridge Biological Technology Company, Beijing, China) for 1 h at room temperature, the immunoblotting signals were visualized using a Western Luminescent Detection kit (Vigorous Biotechnology, Beijing, China).

### TUNEL assay

Apoptosis was determined using TUNEL assay as previously described^19^. Sections were incubated with TUNEL reaction mixture (11684817910, Roche Applied Science, Penzberg, Germany) according to the manufacturer’s instructions. The sections were viewed and analyzed using an Eclipse 80i fluorescence microscope (Nikon, Tokyo, Japan). Ten visual fields were randomly selected and observed under a magnification of 200×.

### Flow cytometry

To identify features of apoptosis in PMVECs, all cells were immunostained according to the protocol of the Annexin V/PI apoptosis kit. The apoptosis was analyzed using flow cytometry.

### Semi-quantitative analysis of ALI in mice

ALI was scored according to the previous description^20^ as follows: (i) alveolar congestion, (ii) hemorrhage, (iii) infiltration or aggregation of neutrophils or macrophages in airspace or vessel wall, and (iv) thickness of alveolar wall or formation of hyaline membrane. A 5-point scale was given on each item as follows: 0 score, minimal damage; 1 score, mild damage; 2 scores, moderate damage; 3 scores, severe damage; 4 scores, maximal damage.

### Additional methods

For the additional methods, the expression of malondialdehyde (MDA) content, the weight-to-dry weight (W/D) ratio, and superoxide dismutase (SOD) in lung were determined according to the previous description^18^. All the tests were carried out at least in triplicate.

### Statistical analysis

Quantitative data was presented as the mean ± standard error of mean, at least from three independent experiments. Statistical differences in mean values were tested by Student’s t-test or by one-way ANOVA using Dunnett’s test in multiple comparisons. Count data was analyzed using Chi-square test. P < 0.05 was considered to be statistically significant.

## Results

### Establishment of an ALI mice model by AngII infusion

Based on the significant elevation of AngII in AAD complicated with ALI patients^3^, ALI model was established through subcutaneous infusion of AngII in this study. The extent of pulmonary injury and PMVEC barrier integrity were determined by evaluating the oxygenation index, pathological morphology, MDA, SOD activity, and W/D ratio. Mice were randomly divided into three groups, including control group (n = 8), Sham group (n = 8), and AngII group (n = 8) through subcutaneous infusion of AngII, respectively.

**Figure 1:**
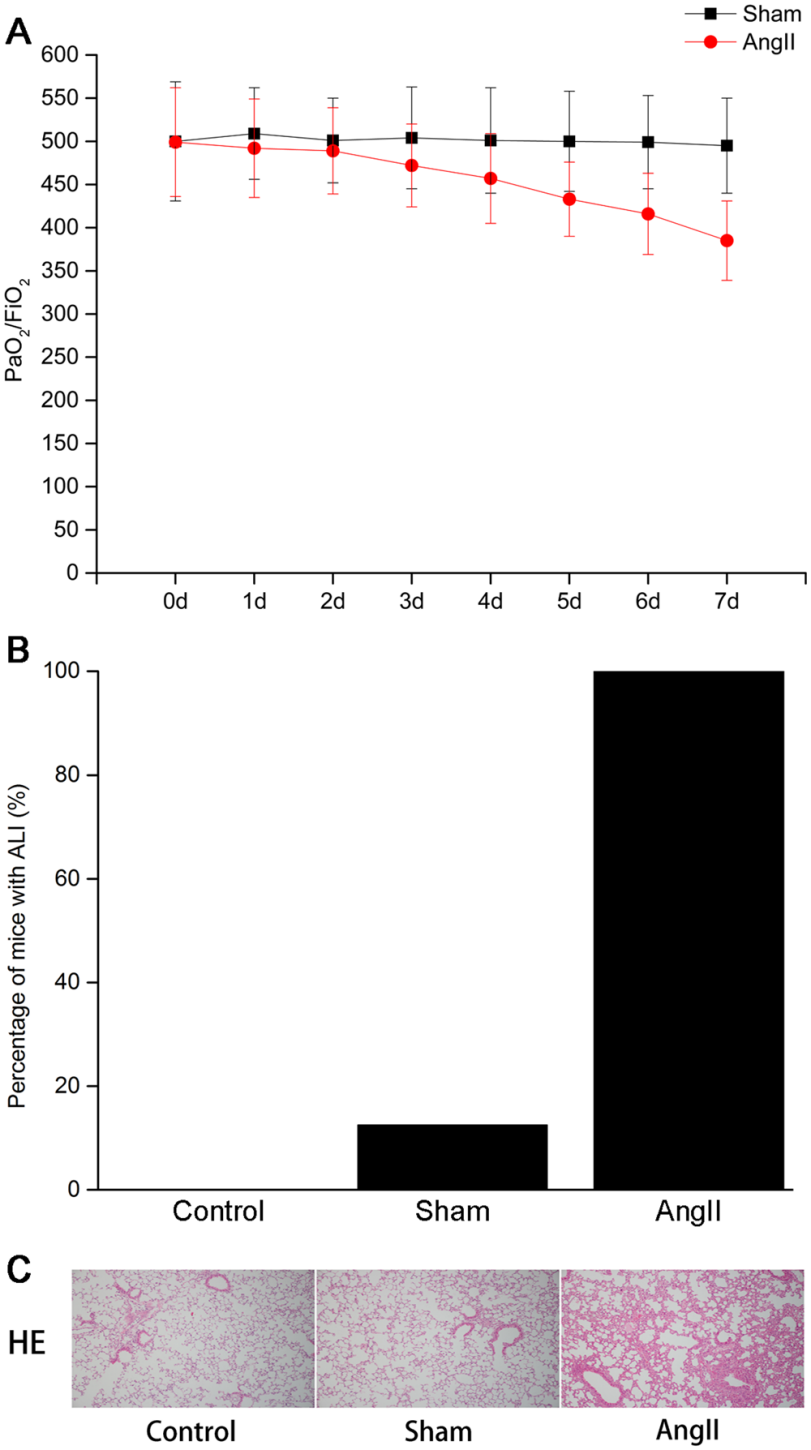
Establishing of mice ALI model through subcutaneous infusion of AngII. (**A**) Blood gas results. (**B**) Comparison of ALI incidence among groups. (**C**) HE staining of the lung tissues in mice. Obvious lung oedema and inflammatory cell infiltration were noticed in the lung tissues in AngII group. The images were observed under a magnification of 100×.

No mortality was noticed in the mice subjected to AngII infusion within 1-week observation period. The PaO_2_ showed decrease two days after AngII treatment (Fig. 1A), and ALI was induced in all animals of the AngII group one week after AngII treatment (Fig. 1B). For the HE staining, obvious pulmonary interstitial edema was noticed in the mice model. Meanwhile, massive infiltration of inflammatory cells was noticed in the lung tissues in the AngII group (Fig. 1C).

### IL-22 attenuated ALI induced by Ang II

To investigate the roles of IL-22 in AngII-induced ALI, mice were divided into control group (n = 8) AngII group (n = 8) AngII+IL-22 group (n = 8) and AngII+IL-22+AG490 group (n = 8). IL-22 or AG490 was given via intraperitoneal injection. The activities of JAK2-STAT3 signaling pathway in the pulmonary injury and PMVEC barrier integrity were evaluated.

**Figure 2:**
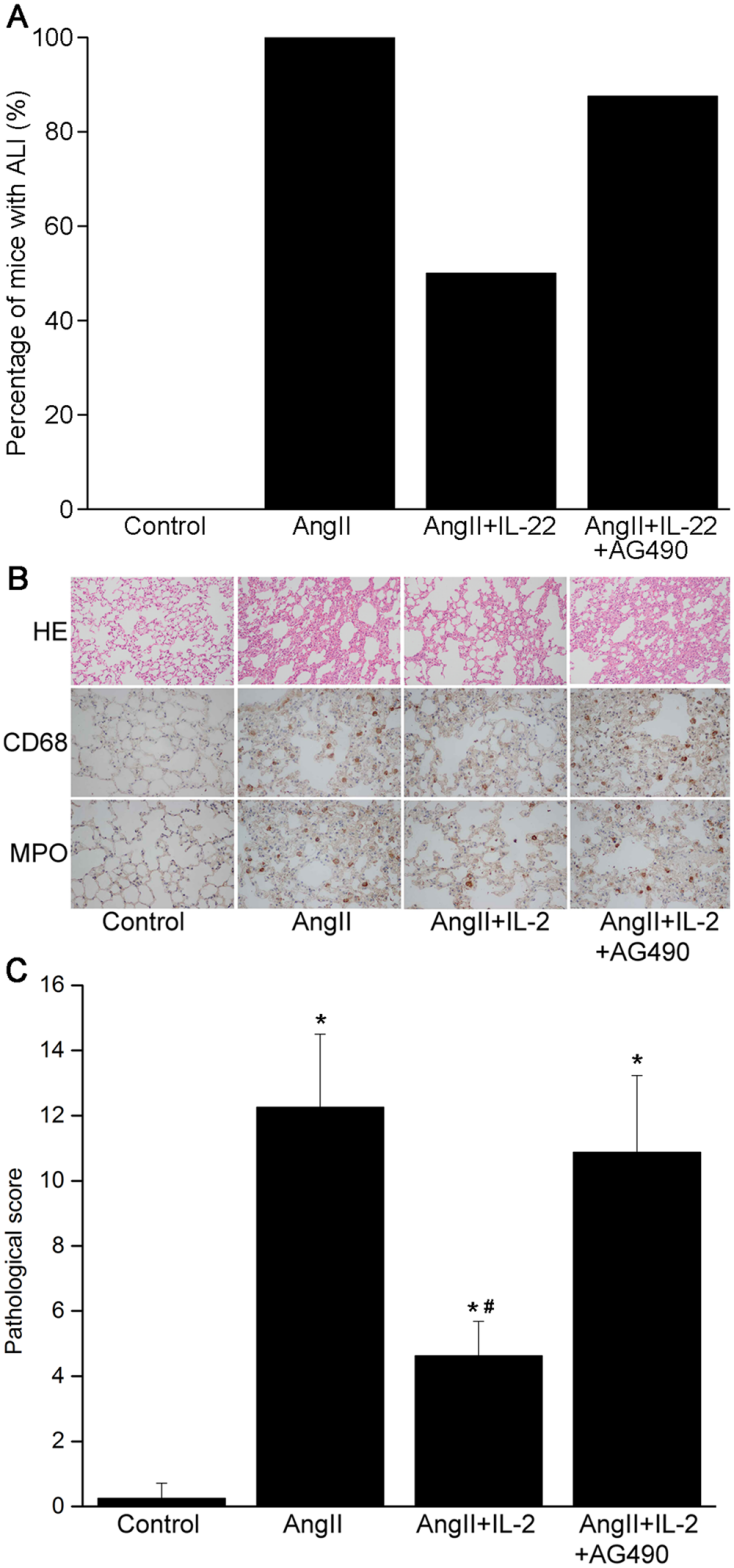
IL-22 attenuated the ALI induced by AngII. (**A**) The incidence of lung injury showed remarkable decrease after IL-22 treatment. (**B**) In the ALI model induced by AngII, obvious oedema was noticed, together with massive infiltration of neutrophils (MPO) and macrophages (CD68), which was significantly reversed after IL-22 treatment. (**C**) IL22 could significantly attenuate the lung injury as revealed by pathological changes, but such phenomenon was completely reserved after AG490. HE staining and immunohistochemistry images were observed under a magnification of 200× and 400×, respectively. ^*^P < 0.05 versus control group. ^#^P < 0.05 versus AngII group and AngII+IL-22+AG490 group.

The incidence of ALI showed obvious decrease in the ALI mice after IL-22 treatment (Fig. 2A). Compared with the AngII group, the pulmonary edema in the AngII+IL-22 group was obviously attenuated, together with decreased infiltration of neutrophils and macrophages (Fig. 2B). IL-22 induced significant attenuation of lung injury in ALI mice **(**Fig. 2C **)**. Meanwhile, the MDA content and W/D ratios in lung showed significant decrease in the AngII+IL-22 group compared with the Ang II group. In contrast, the SOD activity increased significantly in the AngII+IL-22 group compared with the Ang II group (Table 1). However, these phenomenons were reversed after administration of AG490. Taken together, we concluded that IL-22 could attenuate the ALI induced by Ang II, while AG490 could hamper its protective effects.

**Table 1:** Determination of MDA, SOD and W/D.

Group	MDA(nmol/mgprot)	SOD(U/mgprot)	W/D
Control (n = 8)	1.29 ± 0.21	49.26 ± 5.73	3.19 ± 0.48
AngII (n = 8)	4.15 ± 0.58^*^	25.54 ± 3.47^*^	4.97 ± 0.62^*^
AngII+IL22 (n = 8)	2.38 ± 0.41^*,#^	40.57 ± 6.01^*,#^	3.80 ± 0.41^*,#^
AngII+IL22+AG490 (n = 8)	3.13 ± 0.45^*^	31.99 ± 3.72^*^	4.58 ± 0.63^*^

^*^P < 0.05, compared with control group; ^#^P < 0.05, compared with AngII group and AngII+IL22+AG490 group.

### IL-22 inhibited Ang II-induced PMVECs apoptosis through up-regulating the expression of Bcl-2

**Figure 3:**
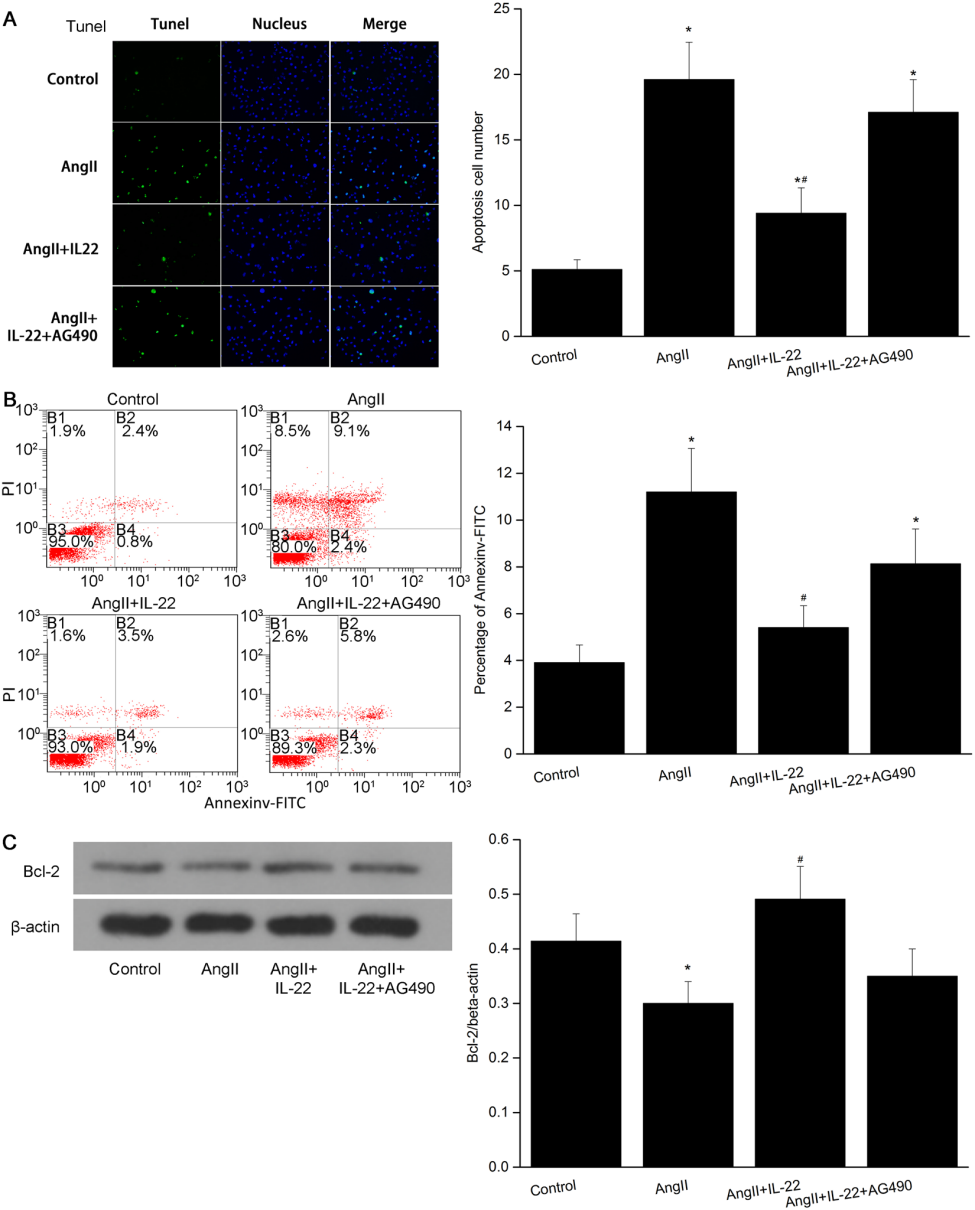
IL22 inhibited the apoptosis of PMVECs induced by AngII. (**A**) IL22 could attenuate the apoptosis of PMVECs significantly as revealed by TUNEL assay, while such phenomenon was completely inhibited after AG490. (**B**) Flow cytometry indicated the apoptosis rate of the PMVECs in the AngII+IL22 group was significantly decreased compared with the AngII group. The apoptosis rate in the AngII+IL22+AG490 group was significantly elevated compared with that of AngII+IL22 group. The raw data were listed in the Supplementary file. (**C**) The expression of Bcl-2 in the PMVECs significantly decreased after AngII stimulation, while the expression of Bcl-2 in the AngII+IL22 group was higher than that of AngII group. ^*^P < 0.05 versus control group. ^#^P < 0.05 versus AngII group and AngII+IL-22+AG490 group. Immunofluorescence images were observed under a magnification of 200×.

Our previous study showed Ang II-induced PMVECs apoptosis was a major cause for the ALI^3^. In this study, we found IL-22 could significantly decrease the incidence of pulmonary injury and attenuate the pulmonary injury. To determine whether IL-22 could inhibit the Ang II-induced PMVECs apoptosis, the animals were divided into control group, AngII group, AngII+IL-22 group, and AngII+IL-22+AG490 group, respectively. Then we determined the apoptosis of PMVECs in each group. Compared with the Ang II group, the apoptotic rate in the Ang II+IL-22 group showed significant decline (Fig. 3A and B), indicating IL-22 showed significant inhibiting effects on Ang II-induced PMVECs apoptosis. However, after administration of AG490, such effect was significantly inhibited, implying that JAK2 played a crucial role in this biological process. Moreover, the expression of Bcl-2 in the Ang II+IL-22 group significantly increased compared with the Ang II group (Fig. 3C). This confirmed that Bcl-2 involved in the anti-apoptosis effects of IL-22.

### Effects of IL22 on STAT3 expression in lung

**Figure 4:**
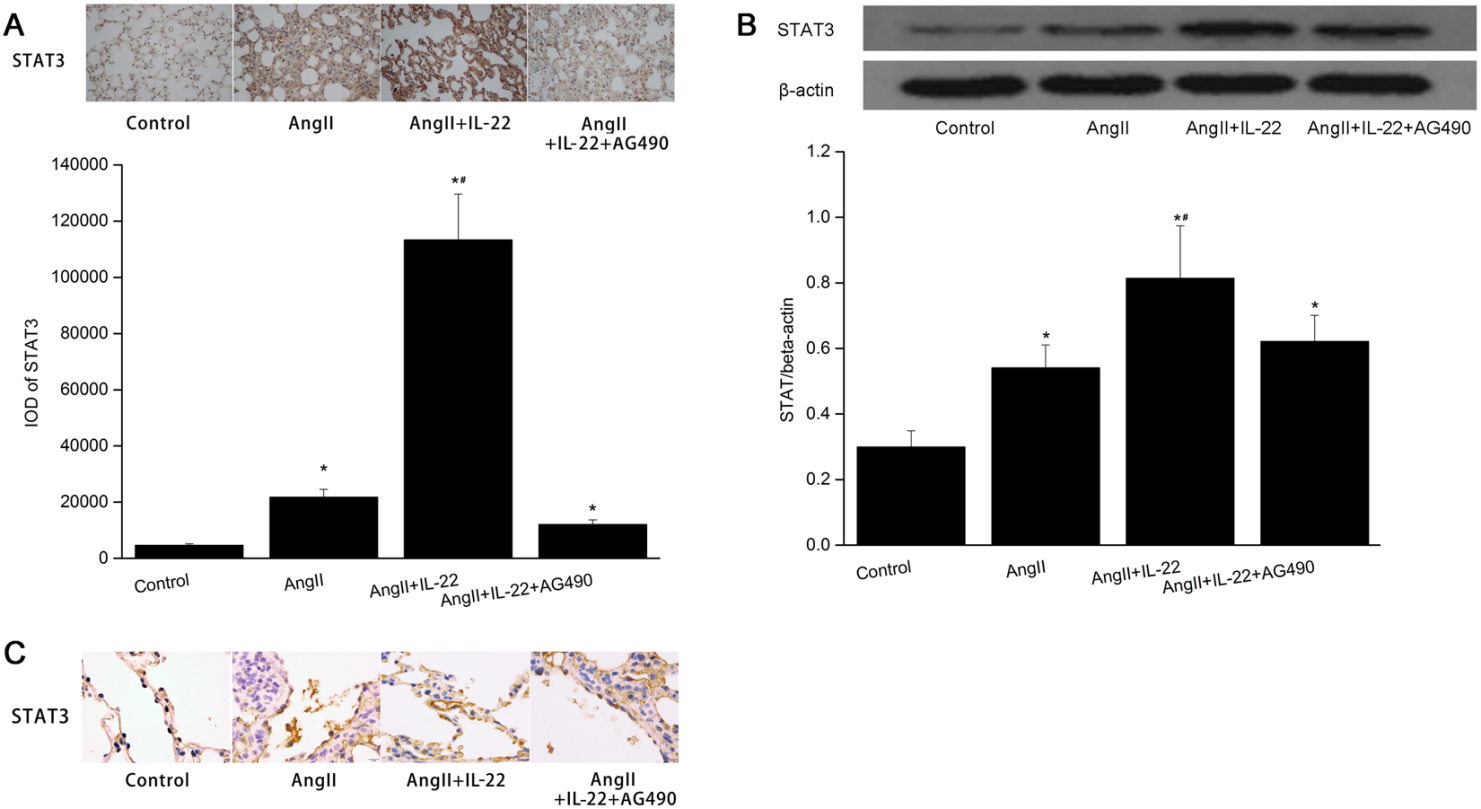
IL22 contributed to the expression of STAT3 in the lung tissues in mice. Immunohistochemistry (**A**, **B**) indicated IL22 induced up-regulation of STAT3 in the lung tissues, but such fact was inhibited after AG490. The raw data were listed in the Supplementary file. (**C**) Western blot analysis indicated the expression of STAT3 in the lung tissues in the AngII+IL22 group was higher than the other groups. ^*^P < 0.05 versus control group; ^#^P < 0.05 versus AngII group and AngII+IL-22+AG490 group. The images of immunohistochemistry were observed under a magnification of 400× and 1000×, respectively.

To evaluate the effects of IL-22 on STAT3 expression in pulmonary tissues in mice, lung sections were immunolabeled for STAT3 1 week after intraperitoneal injection of IL-22. Western blotting and immunohistochemisty analysis indicated that the expression of STAT3 increased in the pulmonary tissues in mice after Ang II stimulation. After IL-22 interference, the expression of STAT3 was significantly up-regulated compared with control group and Ang II group, respectively. Nevertheless, the expression of STAT3 significantly decreased in the Ang II+IL-22+AG490 group compared with the Ang II+IL-22 group (Fig. 4A and C). In the mice lung tissues, IL-22 contributed to the expression of STAT3 in the PMVECs (Fig. 4B).

### Effects of IL22 on STAT3 expression in PMVECs

**Figure 5:**
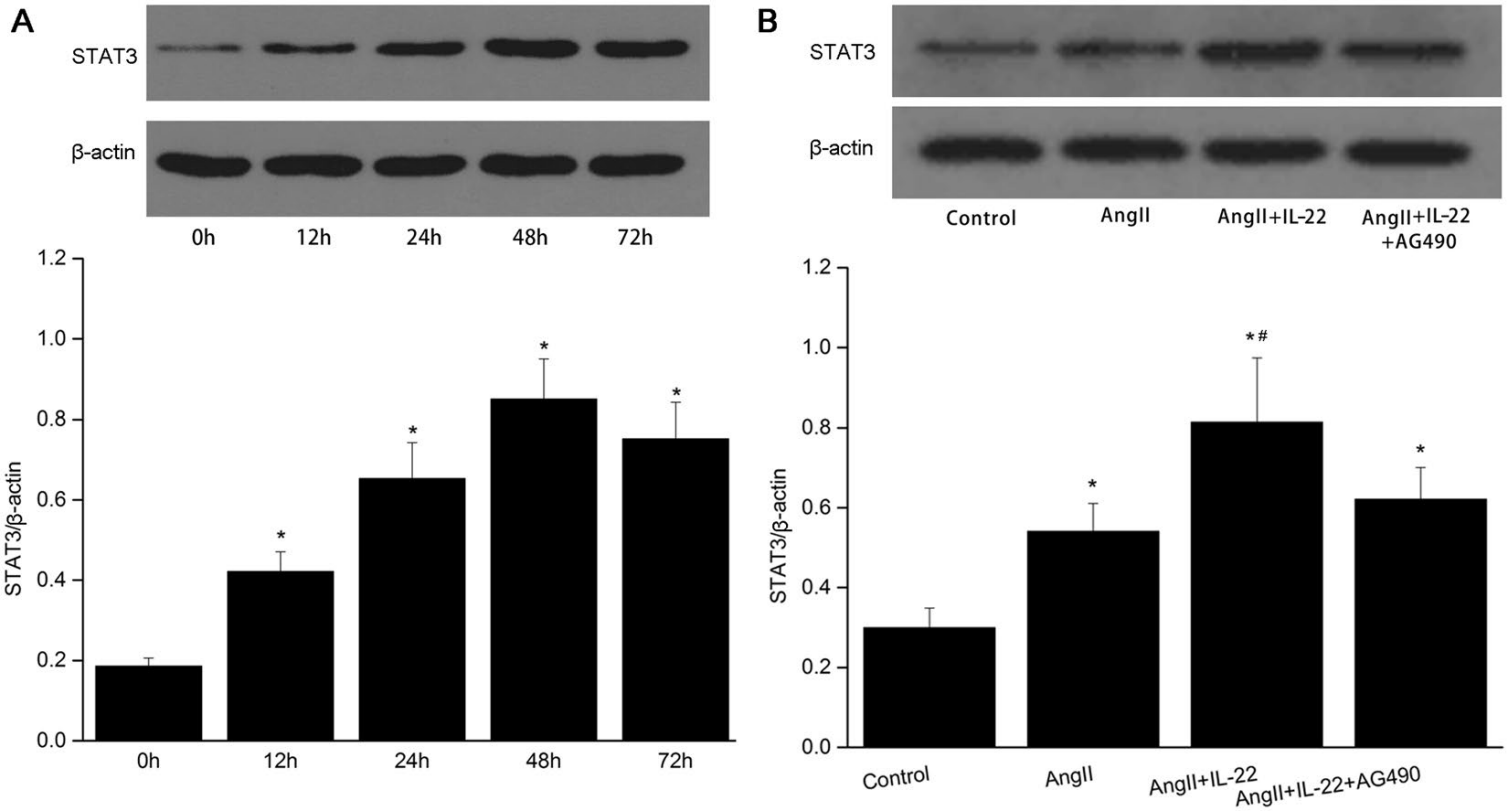
IL22 contributed to the expression of STAT3 in the PMVECs. (**A**) IL22 induced up-regulation of STAT3 in a time-dependent manner, and reached the peak level at 48 h. (**B**) The expression of STAT3 in the AngII+IL22 group was higher than the other groups. The raw data were listed in the Supplementary file. ^*^P < 0.05 versus control group; ^#^P < 0.05 versus AngII group and AngII+IL-22+AG490 group.

To evaluate the effects of IL-22 on PMVECs STAT3 expression, changes of PMVECs STAT3 protein were examined in the control group, AngII group, AngII+IL-22 group, and AngII+IL-22+AG490 group, respectively. As shown in Fig. 5A, the expression of STAT3 in the IL-22 treated PMVECs was up-regulated in a time-dependent manner between 0 h and 72 h, and reached the peak at 48 h. Compared to the control and AngII groups, the expression of STAT3 was significantly up-regulated in AngII+IL-22 group, however, such up-regulation was inhibited after AG490 treatment (Fig. 5B). This indicated that JAK2 was closely related to the elevation of STAT3 induced by IL-22.

### Effects of IL22 on STAT3 phosphorylation in PMVECs

**Figure 6:**
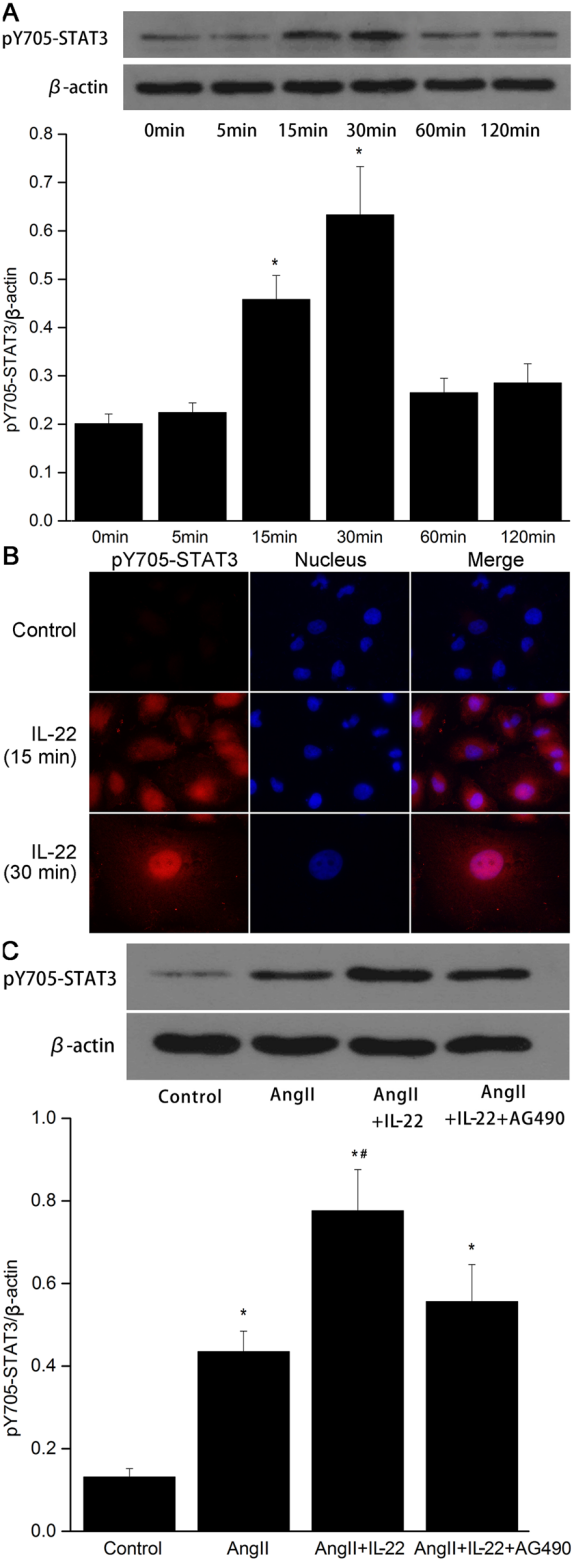
IL-22 significantly stimulated phosphorylation at tyrosine 705 of STAT3 in PMVECs. (**A**) The expression of pY705-STAT3 was up-regulated after IL22 interference, and reached the peak level at 30 min. (**B**) Immunofluorescence analysis indicated the expression of pY705-STAT3 significantly increased together with accumulation in the nucleus after IL22 interference. (**C**) The expression of pY705-STAT3 in the AngII+IL22 group was higher than the other groups. The raw data of Western blot were listed in the Supplementary file. ^*^P < 0.05 versus control group; ^#^P < 0.05 versus AngII group and AngII+IL-22+AG490 group.

Phosphorylation of the STAT3 protein at tyrosine 705 was indicative of kinase activity^21^. In this study, we determined whether STAT3 was activated in response to IL-22, after stimulating by IL-22 (20 ng/mL) at different time points in PMVECs. As shown in Fig. 6A, IL-22 significantly stimulated phosphorylation at tyrosine 705 of STAT3 in PMVECs. The expression of pY705-STAT3 was significantly up-regulated between 15 min and 30 min after treating by IL-22. Immunofluorescent analysis indicated the expression of pY705-STAT3 in PMVECs was extremely lower in control group, while the expression of pY705-STAT3 was accumulated around the nucleus 15 min after IL-22 interference. Meanwhile, most of the pY705-STAT3 was accumulated in the nucleus at 30 min (Fig. 6B). To investigate the effects of JAK2 on the phosphorylation of tyrosine 705 of STAT3 mediated by IL-22, cells were divided into four groups, including (i) control group; (ii) AngII group; (iii) AngII+IL-22 group, and (iv) AngII+IL-22+AG490 group. The phosphorylated tyrosine 705 of STAT3 induced by IL-22 was inhibited after treating with AG490 (Fig. 6C).

### Effects of IL22 on nuclear STAT3 expression in PMVECs

**Figure 7:**
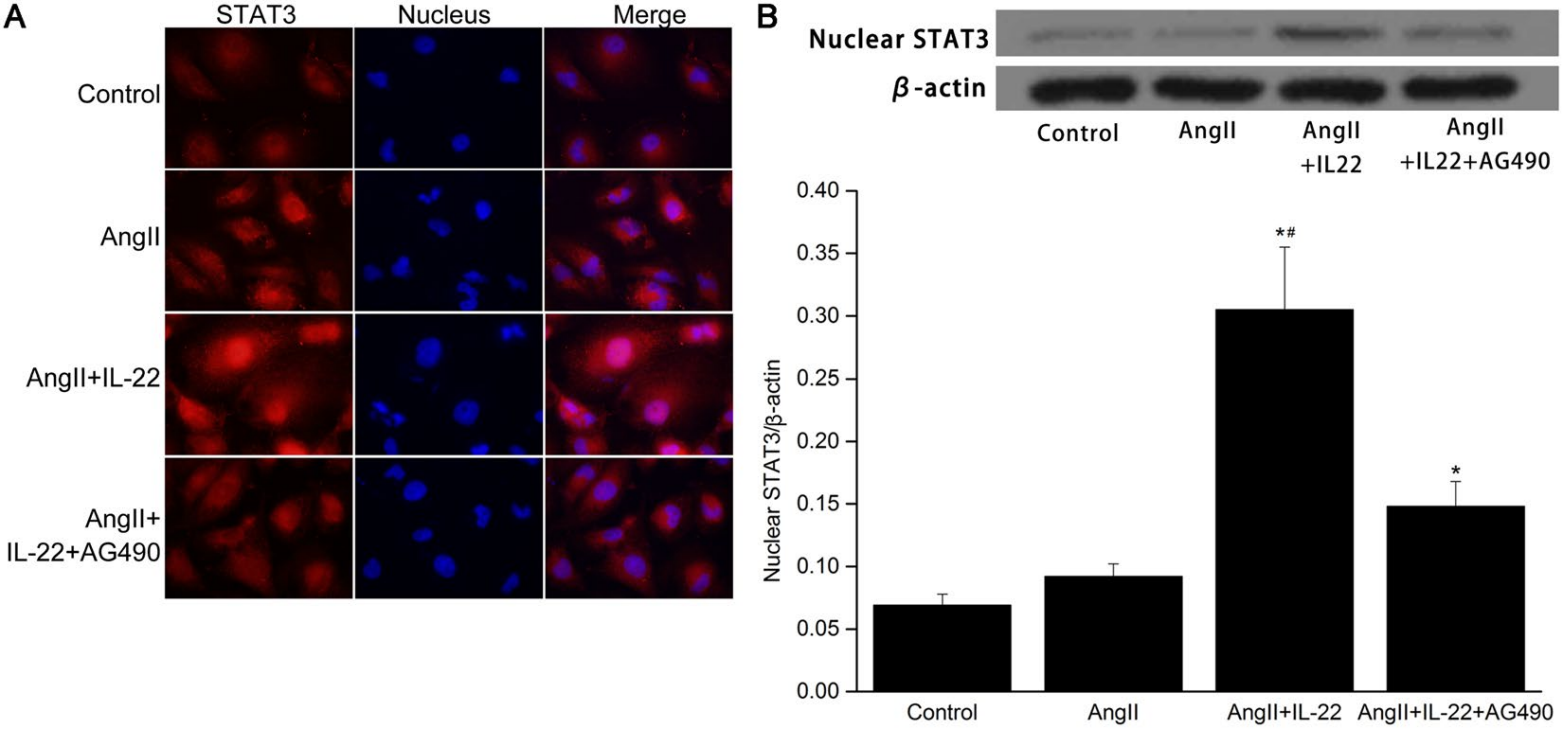
IL-22 significantly stimulated nuclear STAT3 expression in PMVECs. (**A**) STAT3 was mainly localized in the cytoplasm in the control group and AngII group. The expression of STAT3 in the nucleus in the AngII+IL22 group significantly increased, while its expression in the nucleus significantly decreased in the AngII+IL22+AG490 group. (**B**) Western blot analysis indicated the STAT3 in the nucleus in the AngII+IL22 group was significantly higher than the other groups. The raw data were listed in the Supplementary file. ^*^P < 0.05 versus control group; ^#^P < 0.05 versus AngII+IL-22+AG490 group.

As the nuclear pool of STAT3 has been implicated in antiapoptotic/inflammation effect^22–26^, the subcellular location of STAT3 expression after IL-22 stimulation in PMVECs was analyzed in this study in the following groups: control group; (ii) AngII group; (iii) AngII+IL-22, and (iv) AngII+IL-22+AG490 group. STAT3 staining in the nucleus showed remarkable increase in IL-22-stimulated PMVECs (Fig. 7A). STAT3 protein was significantly up-regulated in the PMVECs exposed to IL-22 (Fig. 7B), while AG490 inhibited IL-22 induced nuclear accumulation of STAT3 in PMVECs.

## Discussion

A majority of AAD patients may present ALI featured by hypoxemia, which severely affects the outcome of the patients. Our previous study showed Ang II-induced PMVECs apoptosis was closely related to the pathogenesis of AAD complicated with ALI^3^. The roles of IL-22 in inflammation, tissue protection, regeneration and antimicrobial defense have been well defined. In this study, we aimed to investigate whether IL-22 could attenuate the AngII-induced ALI through inhibiting the apoptosis of PMVECs.

IL-22 derived from activated T cells and NK cells could specifically target the cells *in vivo*, especially the endothelial cells^27^. Increasing evidence indicates IL-22 plays crucial roles in the pathogenesis of several immune-mediated inflammatory diseases. For example, IL-22 was responsible for the psoriasis through modulating the proliferation of keratinocytes, or the expression of protein or inflammatory chemotactic factors in the acute stages^28, 29^. In the pathogenesis of arthritis in mice, IL-22 was reported to contribute to inflammation through inhibiting the expression of collagen and IgG^30^. Meanwhile, IL-22 was reported to involve in the host defense against the Gram-negative bacterial pneumonia or primary influenza virus infection^31, 32^. Furthermore, Sugimoto *et al*. revealed IL-22 ameliorated intestinal inflammation in a mouse model of ulcerative colitis^33^. Currently, the IL-22 related studies have been mainly focused on the respiratory epithelial cells, in which IL-22 is reported to play protective roles through increasing the antibacterial defense, elevating mucus production, increasing proliferation of cells, up-regulating the production of granulocyte-attracting chemokines, and decreasing the lung fibrosis^34–38^. To our best knowledge, rare studies have been carried out to investigate the roles of IL-22 in the cardiovascular system. In 2006, Chang *et al*. showed IL-22 could target the endothelial cells and smooth muscle cells^13^, but its role in the apoptosis is not illustrated. Recently, Shang *et al*. revealed IL-22 derived from endometrial stromal cells (ESCs) contributed to the viability, activation and angiogenesis of HUVEC. In turn, the educated HUVECs may further stimulate proliferation and restrict apoptosis of ESCs, which may result in the progress of adenomyosis. Blocking IL-22 can disturb crosstalk between ESCs and vascularendothelial cells (VECs)^14^. In this study, IL-22 could attenuate the pulmonary edema and massive infiltration of inflammatory cells mediated by Ang II. According to our previous study, the pulmonary injury was mainly induced by Ang II in terms of contributing to apoptosis of endothelial cells and interruption of endothelial barrier integrity^3^. Based on the *in vitro* study, IL-22 could inhibit the apoptosis of PMVECs.

IL-22 acts via a transmembrane receptor complex that consists of two different subunits (i.e. IL-22R1 and IL-10R2) like the other members of IL-10 family^39–41^. The binding of IL-22 and its receptor complex was mainly based on the JAK-STAT signaling pathway. In addition to the JAK-STAT pathway, the activation of mitogen-activated protein kinase (MAPK) pathways may lead to phosphorylation of extracellular signal-regulated kinase 1 (ERK1), ERK2, JUN N-terminal kinase (JNK) and p38 MAPK, together with other pathways including the activation of phosphoinositide 3-kinase (PI3K), AKT and mammalian target of rapamycin (mTOR)^22, 28, 42–45^. However, little is known about the signal pathways involving in the inhibiting effects of PMVECs apoptosis mediated by IL-22.

Our results indicated that IL-22 up-regulated the expression of STAT3 in PMVECs and contributed to the phosphorylation, as well as the intranuclear transmission. After stimulation of AG490, an inhibitor of JAK2 signaling pathway, the STAT3 expression and phosphorylation of Y705-STAT3 was completely inhibited, and migration of STAT3 to the nucleus showed sharp decrease. Meanwhile, the anti-apoptosis of PMVECs and pulmonary protection was significantly attenuated. On this basis, we speculated that IL-22 might be associated with the activation of STAT3 through modulating the JAK2-STAT3 signaling pathway. For the molecular mechanism of IL-22 in the activation of STAT3 in endothelial cells, dimerization may be induced upon the binding between IL-22 and the receptors (Il-22R1 and IL-10R2)^46^, which resulted in the approaching and mutual activation of the JAK kinase linked to the receptor^47^. Upon JAK2 activation, phosphorylation may occur in the Tyr residues, which subsequently leads to formation of docking sites in the Tyr and the peripheral amino acids. On this basis, the STAT3 protein containing a SH2 domain was recruited to this docking site^23^. Subsequently, JAK2 kinase triggered the phosphorylation of STAT3 protein, followed by separation of phosphorylated STAT3 from the IL-22 receptor, which combined with each other between the 608 Arg and the phosphorylated Tyr 705 to form a dimer in cytoplasm finally migrated to the nucleus^47^. In addition to JAK2 kinase, IL-22 may activate the STAT3 through other tyrosine kinases as AG490 could not block the function of IL-22 completely. After migrating to the nucleus, STAT3 could bind specifically with target genes, which initiated the gene expression such as *cyclin D1*, *c-myc*, *c-Jun*, *Bcl-2* and *Bcl-xL* ^22–26^. For the apoptosis of PMVECs, AngII contributed to the down-regulation of Bcl-2 and activation of Caspase 3^3^. Our results showed the expression of Bcl-2 with several binding sites of STAT3^48^ was significantly increased upon STAT3 activation by IL-22.

In conclusion, IL22 showed protective effects on lung injury through inhibiting the AngII-induced PMVECs apoptosis and PMVEC barrier injury. Such process was associated with the activation the JAK2/STAT3 signaling pathway.

## Electronic supplementary material

Supplementary Dataset 1
