# Supplementary material for: Interleukin 22 attenuated angiotensin II induced acute lung injury through inhibiting the apoptosis of pulmonary microvascular endothelial cells
Source: Sci Rep. 2017 May 19;7:2210. doi: 10.1038/s41598-017-02056-w (PMC5438354; doi:10.1038/s41598-017-02056-w)
Supplement: Supplementary file 1 — Supplementary Dataset 1 [file 41598_2017_2056_MOESM1_ESM.doc]

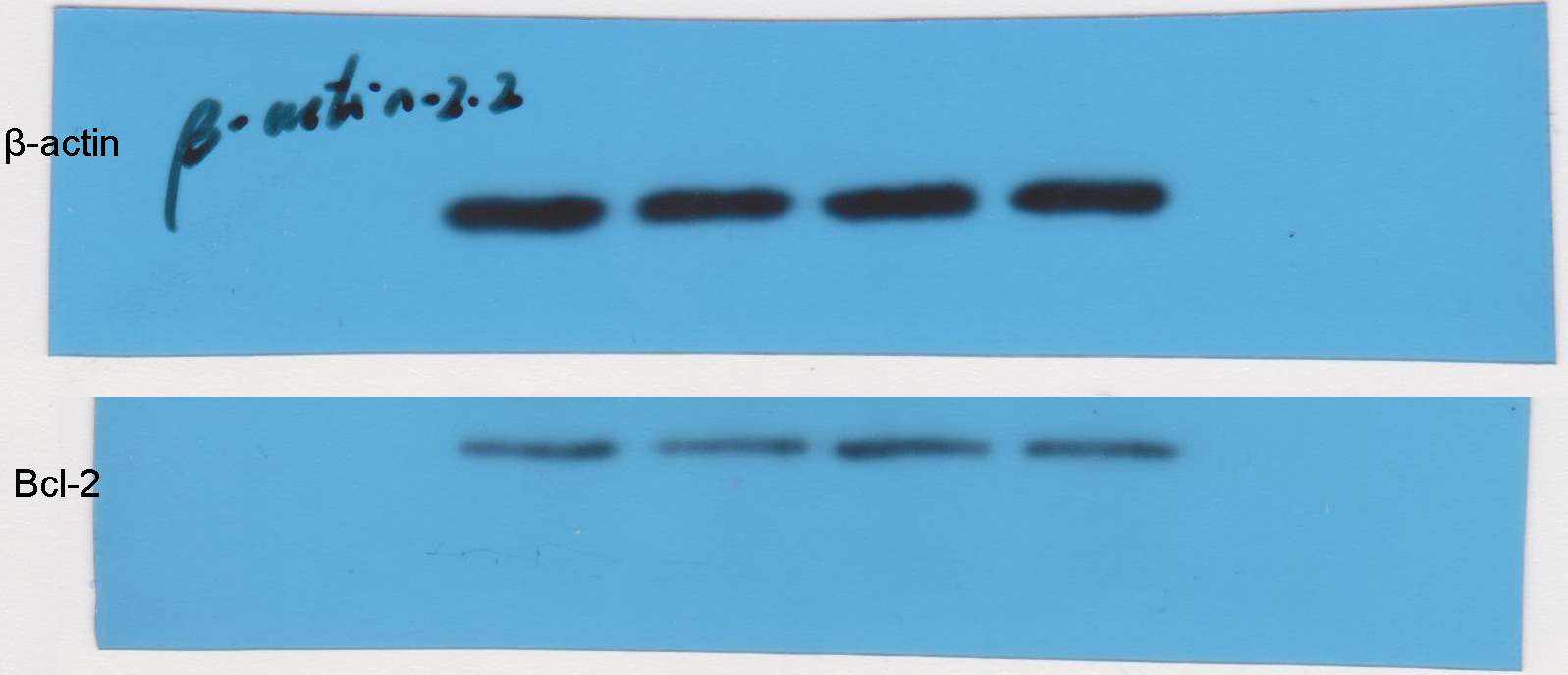


Supplementary figure 1


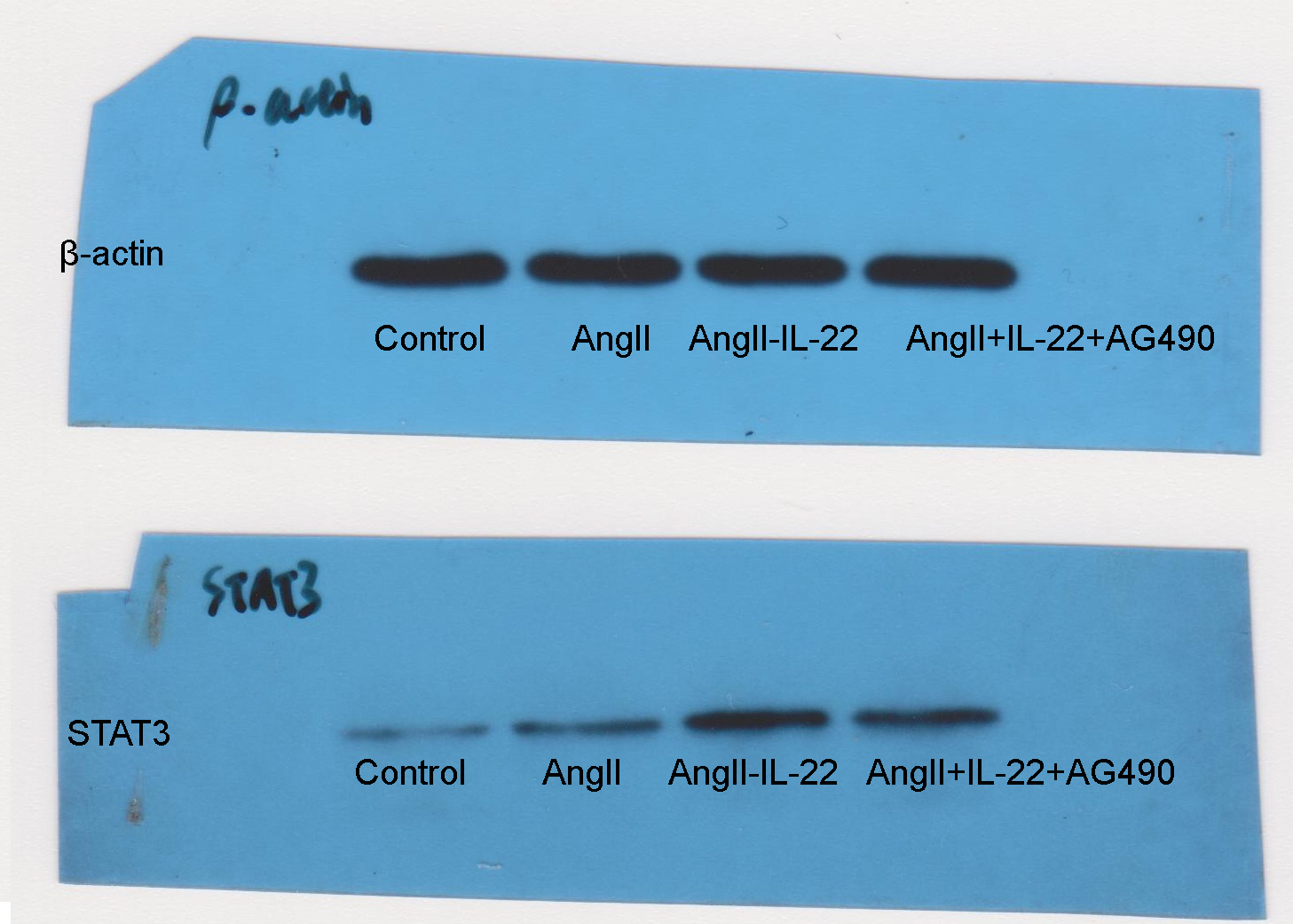


Supplementary figure 2


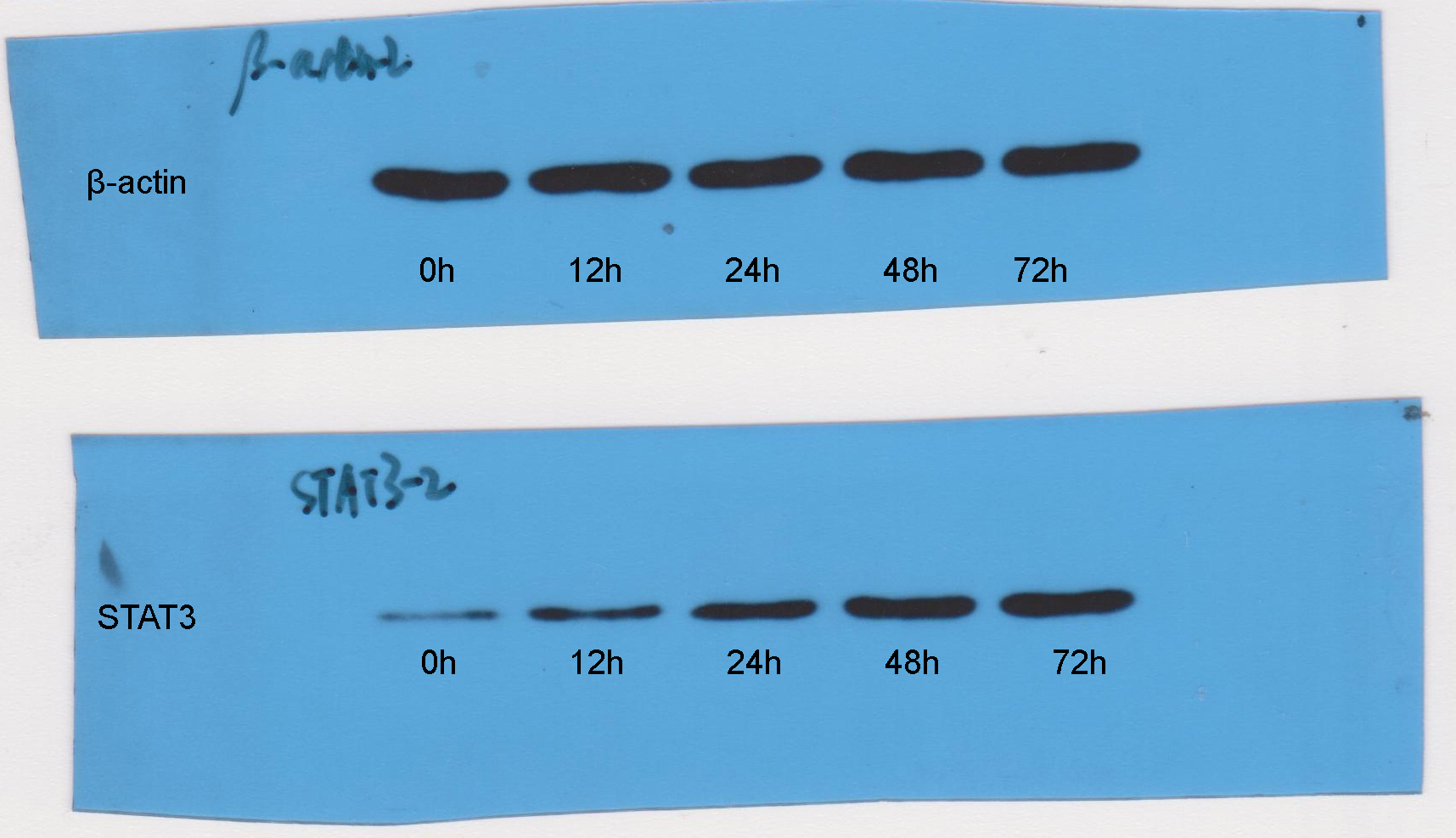


Supplementary figure 3


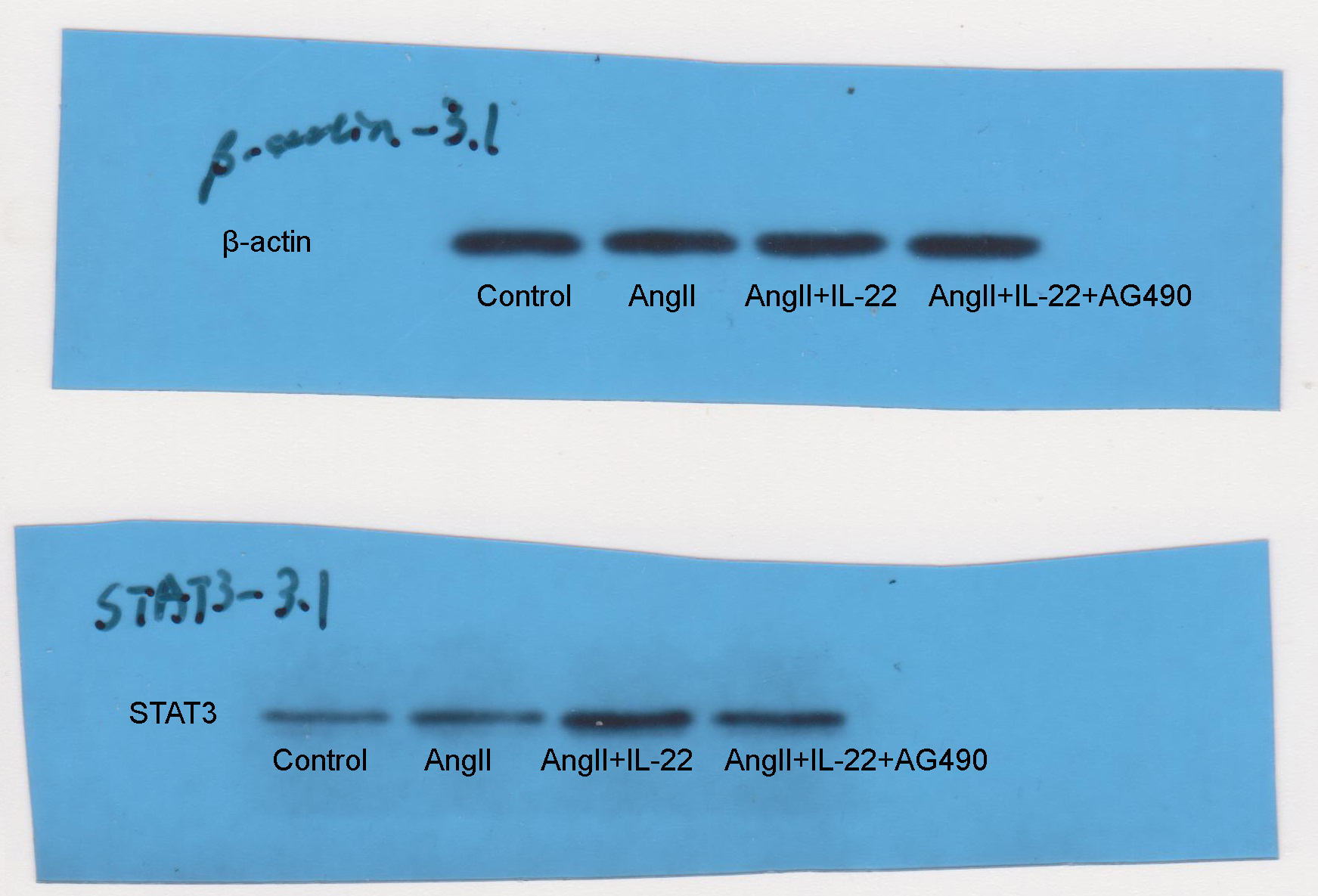


Supplementary figure 4


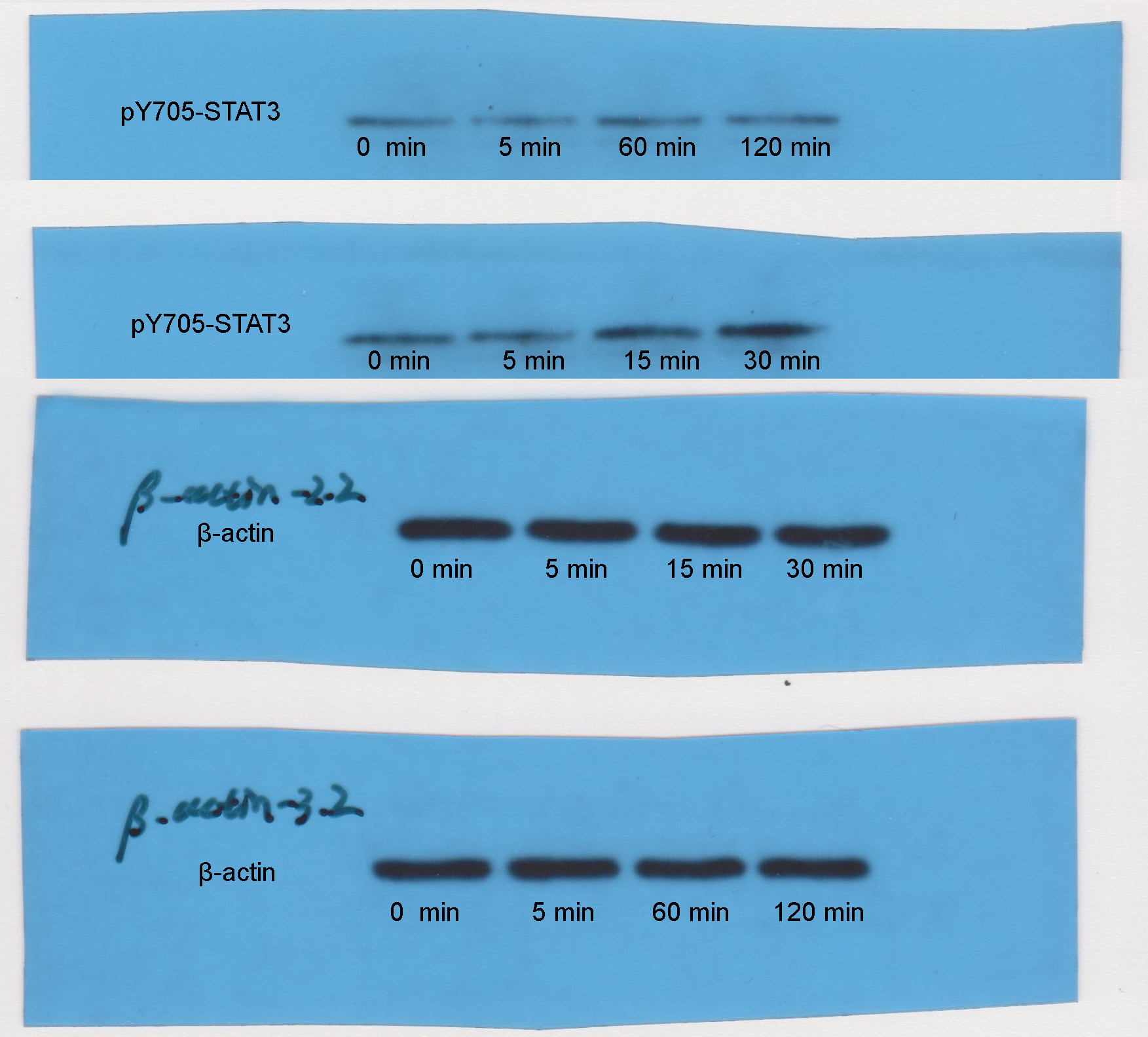


Supplementary figure 5


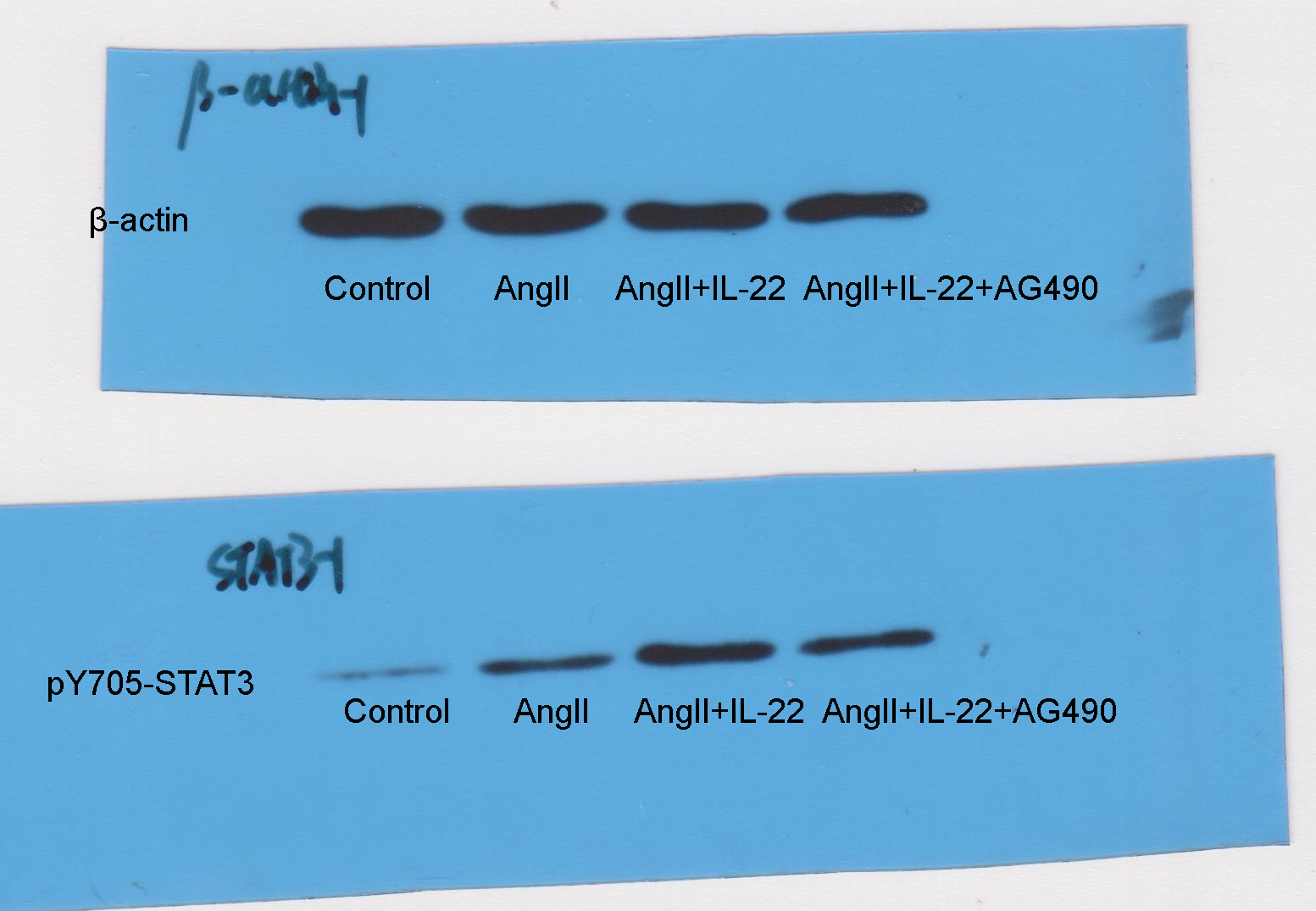


Supplementary figure 6


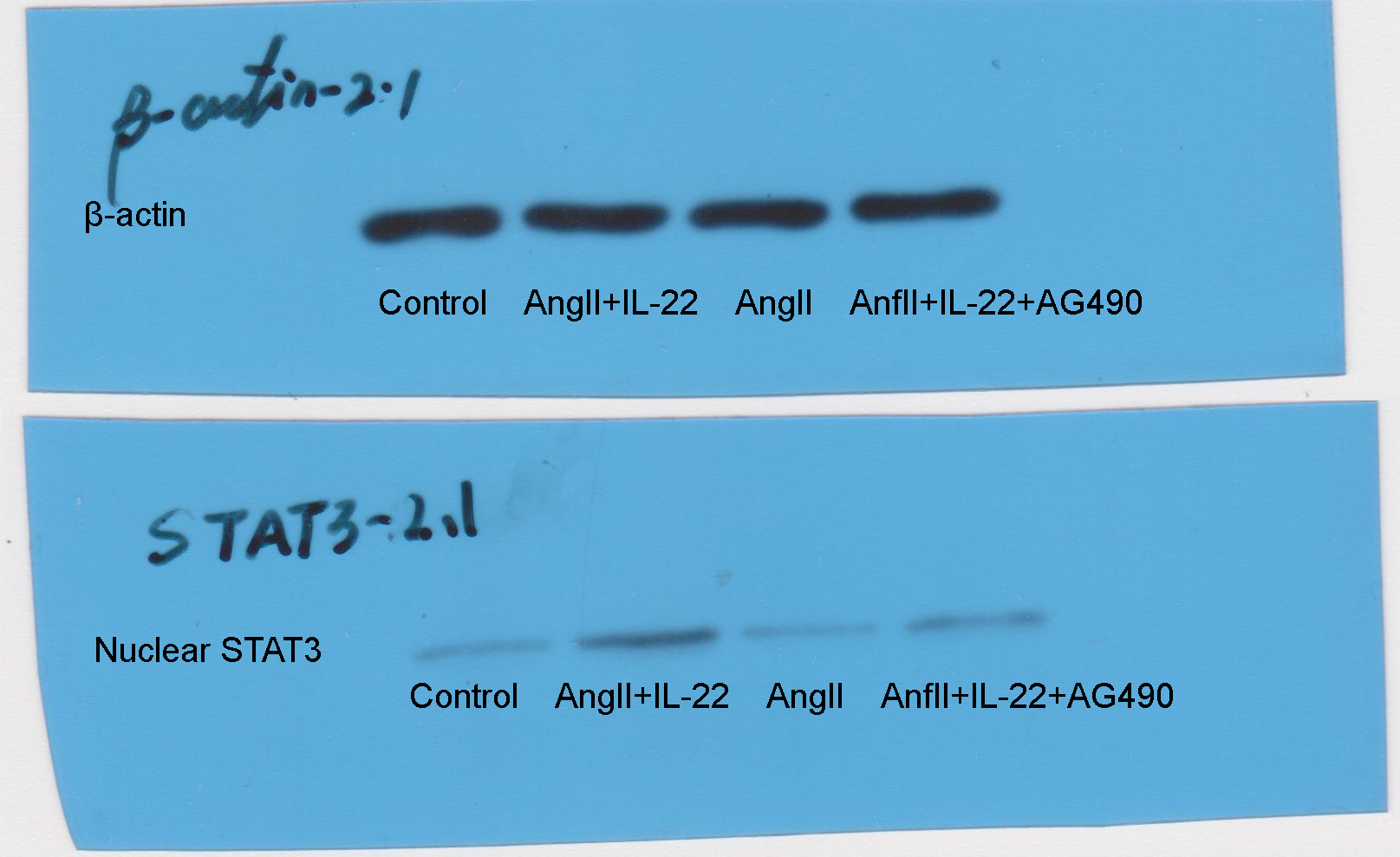


Supplementary figure 7
